# CLUEY enables knowledge-guided clustering and cell type detection from single-cell omics data

**DOI:** 10.1093/bioinformatics/btaf528

**Published:** 2025-09-22

**Authors:** Daniel Kim, Carissa Chen, Lijia Yu, Jean Yee Hwa Yang, Pengyi Yang

**Affiliations:** Computational Systems Biology Unit, Children’s Medical Research Institute, Faculty of Medicine and Health, University of Sydney, Westmead, NSW 2145, Australia; Sydney Precision Data Science Centre, University of Sydney, Sydney, NSW 2006, Australia; Charles Perkins Centre, University of Sydney, Sydney, NSW 2006, Australia; Computational Systems Biology Unit, Children’s Medical Research Institute, Faculty of Medicine and Health, University of Sydney, Westmead, NSW 2145, Australia; Sydney Precision Data Science Centre, University of Sydney, Sydney, NSW 2006, Australia; Charles Perkins Centre, University of Sydney, Sydney, NSW 2006, Australia; Computational Systems Biology Unit, Children’s Medical Research Institute, Faculty of Medicine and Health, University of Sydney, Westmead, NSW 2145, Australia; Sydney Precision Data Science Centre, University of Sydney, Sydney, NSW 2006, Australia; School of Mathematics and Statistics, Faculty of Science, University of Sydney, Sydney, NSW 2006, Australia; Sydney Precision Data Science Centre, University of Sydney, Sydney, NSW 2006, Australia; Charles Perkins Centre, University of Sydney, Sydney, NSW 2006, Australia; School of Mathematics and Statistics, Faculty of Science, University of Sydney, Sydney, NSW 2006, Australia; Computational Systems Biology Unit, Children’s Medical Research Institute, Faculty of Medicine and Health, University of Sydney, Westmead, NSW 2145, Australia; Sydney Precision Data Science Centre, University of Sydney, Sydney, NSW 2006, Australia; Charles Perkins Centre, University of Sydney, Sydney, NSW 2006, Australia; School of Mathematics and Statistics, Faculty of Science, University of Sydney, Sydney, NSW 2006, Australia

## Abstract

**Motivation:**

Clustering is a fundamental task in single-cell omics data analysis and can significantly impact downstream analyses and biological interpretations. The standard approach involves grouping cells based on their gene expression profiles, followed by annotating each cluster to a cell type using marker genes. However, the number of cell types detected by different clustering methods can vary substantially due to several factors, including the dimension reduction method used and the choice of parameters of the chosen clustering algorithm. These discrepancies can lead to subjective interpretations in downstream analyses, particularly in manual cell type annotation.

**Results:**

To address these challenges, we propose CLUEY, a knowledge-guided framework for cell type detection and clustering of single-cell omics data. CLUEY integrates prior biological knowledge into the clustering process, providing guidance on the optimal number of clusters and enhancing the interpretability of results. We apply CLUEY to both unimodal (e.g. scRNA-seq, scATAC-seq) and multimodal datasets (e.g. CITE-seq, SHARE-seq) and demonstrate its effectiveness in providing biologically meaningful clustering outcomes. These results highlight CLUEY on providing the much-needed guidance in clustering analyses of single-cell omics data.

**Availability and implementation:**

CLUEY package is freely available from https://github.com/SydneyBioX/CLUEY.

## 1 Introduction

Advancements in single-cell omics technologies, especially their progression toward multimodality, have transformed our ability to profile global molecular attributes at single-cell resolution. Clustering is a critical task in single-cell omics data analysis, serving as a foundational step for detecting putative cell types before downstream analyses such as differential expressed gene identification and cell type annotation (Heumos *et al.* 2023). The standard approach involves first grouping cells based on their molecular attributes, such as gene expression profiles, and then manually annotating each cluster using distinct marker genes identified through differential expression analysis. The number and the quality of the clusters generated by clustering algorithms can have a significant impact on downstream analyses and their biological interpretation (Butler *et al.* 2018). Yet, for a given dataset, substantial differences in clustering results are often observed (Duò *et al.* 2018, Freytag *et al.* 2018). For instance, different clustering methods applied to the same dataset can produce results with substantially different number of clusters (Yu *et al.* 2022). Additionally, factors such as the choices of dimension reduction techniques, similarity metrics (Kim *et al.* 2019), and clustering algorithm parameters further contribute to the variability in clustering results. These discrepancies often lead to subjective interpretations, complicating downstream analyses such as cell type detection and annotation. To address these challenges, methods such as SC3 attempt to stabilize clustering results by combining multiple clustering methods through a consensus approach (Kiselev *et al.* 2017), whereas methods such as Seurat indirectly achieve clustering consensus by integrating gene expression data with additional data modalities (e.g. surface protein abundance) (Hao *et al.* 2021). Alternatively, various methods have been developed to actively estimate the number of cell types in the data using inherent data structures such as inter- and intra-cluster similarities, eigenvector-based metrics, and clustering stability metrics (Yu *et al.* 2023). Despite these improvements, most clustering algorithms for single-cell data rarely incorporate prior biological knowledge during the clustering process. This omission can result in clusters that do not align with biologically meaningful cell types. Incorporating known biological information, such as known cell type markers, could improve the interpretability of the clustering results and guide the algorithm toward more biologically relevant clusters. This can reduce the risk of generating clusters that are artifacts of the algorithm or driven primarily by statistical patterns rather than biological signals, ultimately improving downstream analyses such as differential expression analysis and cell type detection and annotation.

Here, we propose CLUEY, a knowledge-guided framework for estimating the number of cell types and clustering of both unimodal and multimodal single-cell omics data. Building on concepts from our previous work on optimizing clustering with prior biological knowledge (Yang *et al.* 2015), CLUEY identifies and leverages cell identity genes (Kim *et al.* 2021) associated with a wide range of cell types from large-scale single-cell atlases to construct knowledgebases and subsequently uses them to inform the clustering of query data, determining the most representative number of clusters. This is achieved by recursively clustering until an optimal number of clusters is detected based on the knowledgebase. By integrating prior knowledge, CLUEY performs clustering in an interpretable and biologically-informed manner. Additionally, CLUEY provides interpretable statistics associated with the optimal clustering, facilitating further downstream exploration. We evaluate the performance of CLUEY on both unimodal and multimodal single-cell omics data. Our results demonstrate that CLUEY effectively guides clustering for estimating the number of cell types across a range of datasets with various data characteristics and showcasing its utility in grouping and annotating cells using the knowledgebase. Together, these outcomes highlight CLUEY as a novel route to clustering optimization in single-cell omics analysis.

## 2 Materials and methods

### 2.1 Data collection

#### 2.1.1 Mouse and human cell atlases

The Tabula Muris Atlas (a.k.a. Mouse Cell Atlas [MCA]) (The Tabula Muris Consortium *et al.* 2018) and Tabula Sapiens Atlas (a.k.a. Human Cell Atlas [HCA]) (The Tabula Sapiens Consortium 2022) each comprise two versions: one generated using Fluorescence-Activated Cell Sorting (FACS) and Smart-Seq2 sequencing protocols, and the other using 10x Genomics technology. Lowly expressed genes with less than a total of 10 counts and cells with less than a total of 200 counts were removed from both datasets. Gene expression values were then normalized and log-transformed using the LogNormalize function in Scater (McCarthy *et al.* 2017). Additionally, cell types with fewer than 20 cells were excluded from both atlases in the evaluation.

The MCA (FACS) includes 23 433 genes, 44 779 cells, and encompasses 81 cell types from 20 organs. The MCA (10X) retains the same number of genes but consists of 54 865 cells and includes 55 cell types from 12 organs. Similarly, the HCA (FACS) dataset contains 58 870 genes, 27 051 cells, and encompasses 133 cell types from 24 organs. In contrast, the HCA (10X) dataset, also composed of 32 911 genes, includes 181 775 cells and 132 cell types from 24 organs.

#### 2.1.2 Other independent evaluation datasets

##### 2.1.2.1 Single-cell transcriptomics datasets

We have also included two human and two mouse single-cell transcriptomics datasets (Zeisel *et al.* 2015, Baron *et al.* 2016, Segerstolpe *et al.* 2016, Zilionis *et al.* 2019) to evaluate the method performance on clustering and detecting cell types in these independent datasets.

##### 2.1.2.2 TEA-seq dataset

The processed data of TEA-seq data from measuring PBMC were downloaded from the NCBI Gene Expression Omnibus (GEO) under the accession number GSM5123954, with raw RNA expression and peak accessibility (ATAC) measured for the same cells. We summarized the matrix of ATAC from peak level to gene activity scores using the “CreateGeneActivityMatrix” function in the Seurat v3 package (Hao *et al.* 2021). Genes with fewer than 1% quantifications, including those with zero counts, across cells in each of the two modalities were removed. After preprocessing, we retained 9661 genes in the RNA modality and 16 434 chromatin accessible regions in the ATAC modality, with 6507 cells and nine cell types in both modalities.

##### 2.1.2.3 CITE-seq dataset

This CITE-seq dataset (E-MTAB-10026) measures PBMC from healthy individuals and from COVID-19 patients (Stephenson *et al.* 2021). Only the data from healthy individuals were used in this study. The raw matrices of RNA and ADT and the annotation of cells to their respective cell types from the original study were downloaded from the EMBL-EBI ArrayExpress database under the accession number E-MTAB-10026. RNA and ADT in this dataset were filtered by removing those that expressed in less than 1% of the cells, including those with zero counts, and cell types were filtered by removing those that have less than 10 cells. After filtering, there are 10 665 genes and 192 surface proteins in the RNA and ADT modalities, respectively, including 30 271 cells from 16 cell types.

### 2.2 CLUEY algorithm

**Figure 1.**
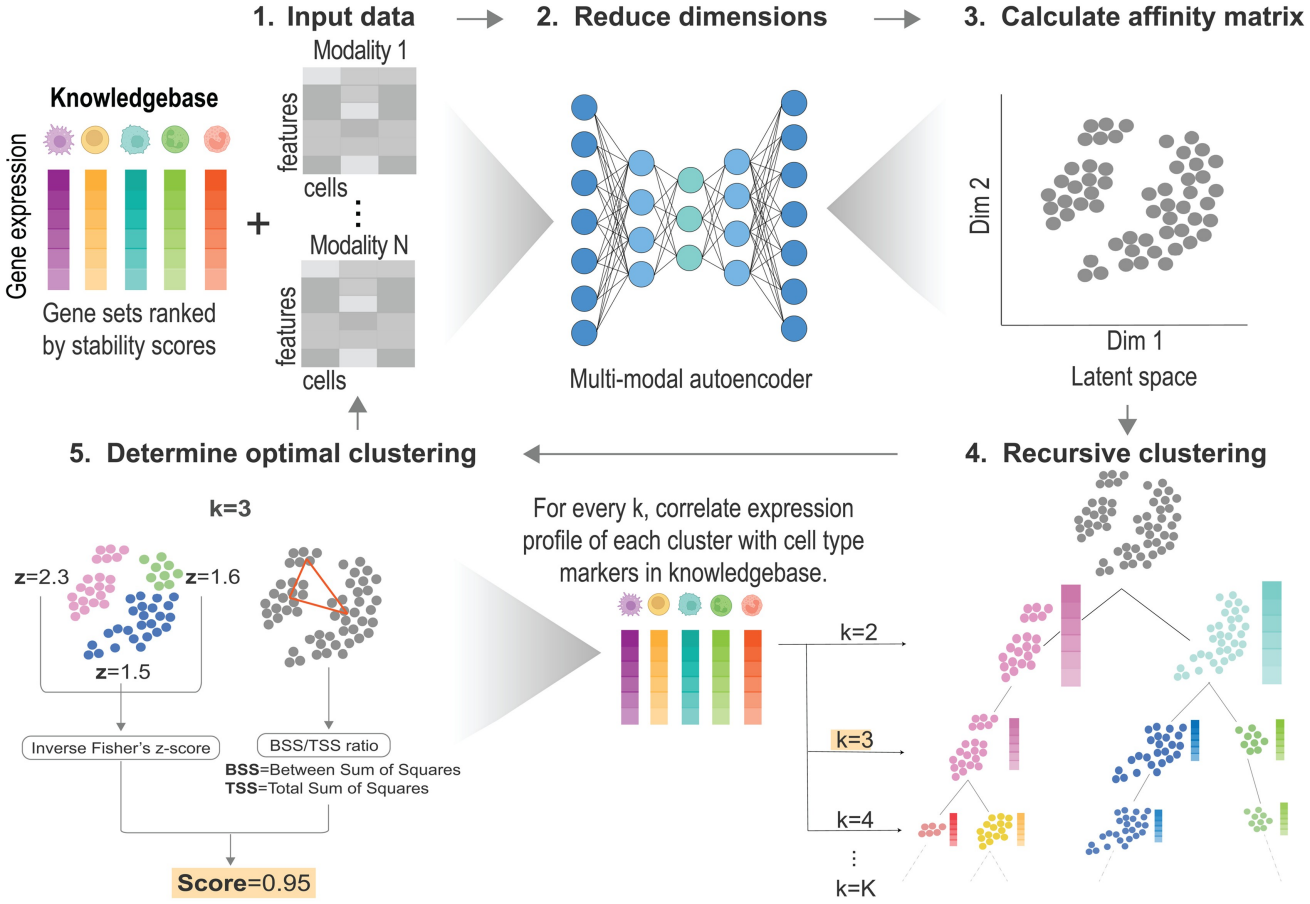
Overview of the CLUEY algorithm. CLUEY builds a knowledgebase of cell types and projects a uni- or multimodal query dataset to a latent space using an autoencoder before calculating an affinity matrix. CLUEY performs clustering using a given clustering algorithm and the knowledgebase to determine the initial optimal number of clusters. The expression profiles of each putative cluster are then averaged and correlated to the gene sets in the knowledgebase. The most correlated cell type in the knowledgebase is used to determine biologically-informed clustering. Step 5: The correlation for each cluster is transformed to a *z*-score using Fisher’s method and averaged using the inverse of Fisher’s method and then combined with the BSS/TSS ratio to form a final score. This is the clustering score for the entire grouping. This process is repeated recursively for each initial cluster identified in Step 4, until an optimal number of clusters is reached.

CLUEY is designed to leverage prior knowledge to guide clustering and is therefore not tied to a particular clustering algorithm. The CLUEY algorithm is composed of three components, excluding the generation of the knowledgebase (Fig. 1). First, given a query dataset, the dimensions of the data are reduced using a multimodal autoencoder (dimensionality reduction). The second component involves the initial estimation of the optimal number of cell types in a uni- or multimodal query dataset, using a knowledgebase. The third and final step is recursive clustering, which recursively clusters the query data to capture finer cell type populations and more thoroughly determine the final prediction of the optimal number of cell types in the data. CLUEY requires gene sets of known cell types to inform the clustering process. We generate sets of cell identity genes for each cell type in a given reference gene expression matrix by differential stability using Cepo (Kim *et al.* 2021). The expression profiles of the cell identity genes are then averaged across the cell types in the knowledgebase to generate their respective pseudo-bulk profiles. For broader usability, we have also implemented the option for creating pseudo-bulk profiles of differentially expressed genes from a knowledgebase that have a Bonferroni corrected *P*-value <.05 for each cell type in the knowledgebase using Seurat. It is also possible to provide a set of any manually determined gene sets and their corresponding averaged expression values as the knowledgebase to CLUEY. Below we give a detailed description of knowledgebase and key components of CLUEY’s algorithm.

#### 2.2.1 Knowledgebase generation

The mouse and human cell atlases were used to create default knowledgebases for CLUEY. Specifically, gene expression matrix and cell type labels from these atlases were used to identify cell identity genes for each type included in these atlases using Cepo R package (Kim *et al.* 2021). Briefly, Cepo selects genes that are differentially stably expressed in each cell type relative to other cell types, under the assumption that genes important for the identity of a cell type are uniquely and stably expressed relative to other cell types. To quantify this, Cepo calculates differential stability statistics for each gene by accounting for the proportions of zeros and coefficient of variation within a cell type and relative to other cell types. The top 50 genes identified by Cepo for each cell type are used as the cell identity genes for that cell type. The knowledgebase is then created by calculating the average profile of each cell type using the union of all cell identity genes and used subsequently by CLUEY to guide the clustering process on a given dataset through estimating the optimal number of clusters in the dataset.

#### 2.2.2 Dimensionality reduction

Let X be a matrix from a single-cell omics data. We select the top 5% highly variable genes (HVGs) from X to subset the matrix and then standardize then it so that each feature has zero mean and unit variance. Building on the framework introduced by Matilda (Liu *et al.* 2023), we next employed a stacked autoencoder to iteratively perform dimension reduction and clustering. First, we encode X into a low-dimensional embedding denoted as Z and reconstruct it by minimizing the mean-squared-error (MSE) loss function


$$L(\theta )=\frac{N}{i=1}\sum_{i=1}^{N}{\left(x_{i}-f(z_{i};\theta )\right)}^{2}$$

where $x_{i}$ is the input profile of a cell, the $f(z_{i};\theta )$ is its reconstruction generated by the decoder network parameterized by θ, and N is the total number of cells. We then extract the latent space Z and construct an affinity matrix for clustering cells. After estimating the optimal number of clusters in the current iteration, we concatenate the latent representation Z with the input matrix corresponding to each identified cluster *X*′ and repeat the dimensionality reduction and clustering steps iteratively. This process of combining the latent space Z with *X*′ is referred to as “stacking” and is a defining characteristic of stacked autoencoders, which occurs during the training process. Stacking allows the model to progressively refine feature representations across multiple layers or iterations, capturing increasingly abstract patterns in the data, without any changes in architecture. Hyperparameters such as the learning rate, number of hidden layers, and encodings can be specified by the user and adjusted according to the needs of the query dataset. We note that the choice of clustering algorithm is independent from the dimension reduction component and is described in detail in the next section.

#### 2.2.3 Clustering optimization for estimating number of clusters

Let to determine the optimal number of clusters in a given query dataset, we cluster the data from *k* = 2 to a user-defined *K* number of clusters (*K* = 20 as the default) via spectral clustering (default). However, users can apply other alternative clustering, such as *k*-means clustering, highlighting that CLUEY’s approach is not exclusively tied to a particular clustering algorithm. For each clustering *k*, we generate pseudo-bulk gene expression profiles for each predicted cluster $C_{i}$ by aggregating the expression values of all cells in the cluster. We then compare each cluster profile to the pseudo-bulk expression profile of each reference cell type or class $R_{j}$ from the knowledge base.

To focus the comparison on informative genes, by default we restrict the analysis to the intersection between the genes in $C_{i}$ and the top 10% most differentially stably expressed genes in $R_{j}$ (this parameter can be adjusted). For each pair cluster $C_{i}$ and reference profile $R_{j}$ we compute the Spearman’s rank correlation coefficient $p_{ij}$, using only the intersecting genes


$$p_{ij}=1-\frac{6\sum_{g=1}^{G}d_{g}^{2}}{G(G^{2}-1)}$$

where G is the number of intersecting genes between the predicted cluster $C_{i}$ and $R_{j}$, and $d_{g}$ is the difference in rank between gene g’s expression in $C_{i}$ and $R_{j}$. This allows for determining the most similar cell type of class in the Knowledgebase to the predicted cluster. Unlike the Pearson’s correlation coefficient, the Spearman’s rank correlation coefficient allows for detecting both linear and non-linear associations. The correlation coefficients $p_{ij}$ are then transformed using Fisher’s Z transformation ($z_{ij}$) for a given cluster i and reference cell type or class j and is written as


$$z_{ij}=w_{i}\frac{1}{2}\ln(\frac{1+p_{ij}}{1-p_{ij}})=\tanh^{-1}\left(w_{i}\times p_{ij}\right)$$

where w represents the weight for cluster i. This weight is defined as the proportion of intersecting genes between the predicted cluster i and cell type or class j in the knowledgebase. It ensures that the contribution of each correlation coefficient $p_{ij}$ is proportional to the number of genes used in its calculation, giving greater influence on associations based on larger sample sizes. Because correlation coefficients are not normally distributed, particularly when they are close to −1 or 1, taking the arithmetic mean of raw correlation coefficients can be statistically inappropriate and lead to biased estimated, hence we apply Fisher’s Z transformation to the weighted correlation coefficients before averaging, as stated above. Via this transformation, we ensure that the resulting scores used to evaluate clustering performance are both statistically valid and comparable across different clusterings. After applying the transformation, we average the *z*-scores for each grouping of clusters (*k* = 1, 2, 3, …*K*) to get $\bar{z}$ and use the inverse of Fisher’s Z transformation to convert the *z*-score $\bar{z}$ back into a Spearman’s rank correlation coefficient for each clustering $p_{k}$, allowing for a more interpretable summary statistic for each clustering


$$p_{k}=\frac{e^{2\bar{z}}-1}{e^{2\bar{z}}+1}=\tanh(\bar{z})$$

In parallel, we also calculate the explained-variance ratio of the between sum of squares (BSS) versus the total sum of squares (TSS) statistics


$$\mathrm{TSS}=\sum_{i=1}^{N}{\left(x_{i}-\bar{x}\right)}^{2};\;\mathrm{BSS}=\;\sum_{j=1}^{K}{n_{j}({\bar{x}}_{j}-\bar{x})}^{2}$$

Here, $\bar{x}$ is the overall mean, $n_{j}$ is the number of cells in cluster j and ${\bar{x}}_{j}$ is the centroid of the jth cluster. We select the kth partition that maximizes the clustering score defined as follow


$$\mathrm{Clustering}\;\mathrm{score}=\frac{\mathrm{BSS}}{\mathrm{TSS}}p_{k}$$

These clusters will then be re-clustered using CLUEY’s recursive clustering algorithm until no less than 30 cells are reached for each cluster. Importantly, the stopping condition can be specified such that the algorithm continues to recluster until no less than a minimum number of cells is specified by the user (default = 30 cells). The final prediction for the optimal number of clusters is then returned, including the respective correlation coefficient and the closest matching reference cell types for interpretability.

### 2.3 Settings for alternative methods

Here, we summarize the settings and parameters used for other methods for comparison and evaluation.

#### 2.3.1 Seurat

We used Seurat (v5.0.2) to process and cluster the query data as outlined in the author’s vignette (https://satijalab.org/seurat) (Hao *et al.* 2021). First, the raw RNA count matrices were used as input and log-transformed using the “NormalizeData” function in Seurat. We used the default setting and thus selected the top 2000 highly variable genes using the “FindVariableFeatures” function before using the “runPCA” function to reduce the dimensions of the data. We then used the “FindNeighbors” and “FindClusters” functions to determine the predicted clusters in the query dataset.

We also ran Seurat on multimodal data with similar steps taken as above. Here, we used the parameters of normalization.method = “CLR” and margin = 2 in the in “NormalizeData” function, as suggested in the author’s pipeline. PCA was performed using the “runPCA” function and the function “FindMultiModalNeighbors” integrates RNA and ADT/ATAC modalities using the PCA results. The joint visualization of RNA and ADT/ATAC were generated using the “wnn.umap” function.

#### 2.3.2 Monocle3

We used the latest version of Monocle3 (v1.3.7) with default parameter settings and followed the pipeline specified by the author (https://cole-trapnell-lab.github.io/monocle3) (Cao *et al.* 2019). Briefly, each dataset was initiated as a Monocle3’s main class “cell_data_set”, and then preprocessed before reducing the dimensions of the data using the “preprocess_cds” and “reduce_dimension” functions, respectively. Cells in the query data were then assigned predicted cluster membership using the “cluster_cells” function.

#### 2.3.3 SC3

We used SC3 (v1.32.0) (Kiselev *et al.* 2017) with default settings as outlined in the author’s tutorial (https://nbisweden.github.io/workshop-archive/workshop-scRNAseq/2018-05-21/labs/sc3_ilc.html). First, we estimated the initial number of cell types in the normalized and log-transformed query data using the “sc3_estimate_k” function. The final predictions were extracted by calling the “sc3” function using the initial estimate as a parameter.

#### 2.3.4 CIDR

Cidr (v0.1.5) (Lin *et al.* 2017) with the default parameters was used on the normalized and log-transformed data to estimate the number of cell types in the query data (https://github.com/VCCRI/CIDR). The query data were converted to an scData object using the “scDataConstructor” function. Dropout candidates and the dissimilarity matrix were calculated using the “determineDropoutCandidates” and “scDissim” functions, respectively, before reducing the dimensions of the data using “scPCA”. Finally, the number of predicted clusters was extracted after running “scCluster”.

#### 2.3.5 RaceID

We used RaceID (v0.3.5) (Grün *et al.* 2015) to acquire the predicted number of clusters in the query data. The query data were first converted to an SCseq object before filtering the data using the “filterdata” function. Distances between the cells were calculated using the “compdist” function. We specified Pearson’s correlation as the distance metric (default) and extracted the predicted clusters using the “clustexp” function with default parameters (https://github.com/dgrun/RaceID3_StemID2_package/blob/master/README.md).

#### 2.3.6 SHARP

The raw counts were provided as input to SHARP (v1.1.0) (Wan *et al.* 2020). Clusters were then predicted using the “SHARP” function, expression type was set to “count” and prep was set to true to preprocess the data and remove any non-expressed genes from the query data.

#### 2.3.7 Spectrum

Spectrum (v1.1) was used with default parameters, where the maximum number of cell types that could be predicted was set to 30 (John *et al.* 2020). Cluster assignments were determined using the “Spectrum” function and raw counts were provided as the query data.

### 2.4 Performance evaluation

We conducted comprehensive testing of CLUEY’s performance against seven methods across four distinct scenarios. Our evaluation focused on assessing CLUEY’s capability to accurately estimate the number of cell types in a query datasets and handle imbalance data. Additionally, we examined CLUEY’s clustering capabilities across four independent datasets, comprising two human and two mouse datasets.

#### 2.4.1 Scenario one

We assessed CLUEY’s ability to annotate and estimate the number of cell types in a query dataset by subsampling the MCA (10X) and HCA (FACS). We excluded cell types with fewer than 200 cells and varied the number of cell types from five to 20 while keeping the number of cells for each cell type constant at 200. Thus, we generated 10 datasets for each specified number of cell types. This resulted in 40 datasets for each atlas.

#### 2.4.2 Scenario two

We investigated the impact of imbalanced data by creating major and minor cell types. Ten cell types were randomly selected from each atlas and categorized into major and minor groups. We adjusted the cell counts within these groups to establish imbalance ratios of 2:1, 4:1, and 8:1 between the major and minor cell types. For instance, a 2:1 ratio indicates 200 cells in major cell types and 100 cells in minor cell types. This process was iterated 10 times for each ratio, yielding a total of 30 datasets.

#### 2.4.3 Scenario three

We evaluated CLUEY on independent datasets, which included two human and two mouse datasets (Zeisel *et al.* 2015, Baron *et al.* 2016, Segerstolpe *et al.* 2016, Zilionis *et al.* 2019). To estimate variability, we randomly subsampled each of the four independent datasets to retain approximately 90% of the original number of cells and repeated this process 10 times, resulting in 40 datasets for each species.

#### 2.4.4 Scenario four

Finally, we evaluated CLUEY on multimodal data using the TEA-seq (Swanson *et al.* 2021) and CITE-seq (Stephenson *et al.* 2021) datasets. In the same manner as the third setting, we down-sampled the data to 90% of the original number of cells and repeated this 10 times to capture variability. This yielded a total of 20 datasets, 10 for each sequencing platform. To assess CLUEY’s ability to cluster a multimodal dataset using a knowledgebase generated unimodal data, we clustered the datasets using the knowledgebase generated from the HCA (10x), which only contains gene expression values (RNA).

### 2.5 Cell clustering evaluation metrics

To assess cluster concordance of CLUEY and the other tested methods, we used three evaluation metrics: adjusted rand index (ARI), and absolute deviation as defined below.

Given a set of *n* elements, and two groupings or vectors of cluster labels, namely $X=\{X_{1},\;X_{2},\;\ldots ,\;X_{n}\}$ and $Y=\{Y_{1},\;Y_{2},\;\ldots ,\;Y_{n}\}$, the overlap between X and Y can be summarized in a contingency table $\left[n_{ij}\right]$ where each entry $n_{ij}$ denotes the number of elements in common between $X_{i}$ and $Y_{j}$. Furthermore, $n_{ij}=|X_{i}\cap Y_{j}|$, $a_{i}=\sum_{j}n_{ij}$, and $b_{j}=\sum_{i}n_{ij}$. Thus, the ARI is defined as


ARI = ∑ij(nij2)-[∑i(ai2)∑j(bj2)](n2)12[∑i(ai2)+∑j(bj2)]-[∑i(ai2)∑j(bj2)]/(n2)

To quantify the difference between the predicted number of clusters versus the true number of clusters, we took the absolute value of their difference and divided by the number of cell types in a dataset to calculate the absolute deviation ratio (ADR) as shown below


$$\mathrm{ADR}=\left|\;c\;-\;\hat{c}\;\right|/c$$

where c is the true number of cell types and $\hat{c}$ is the predicted number of cell types.

To fairly compare CLUEY and the tested methods across all tested scenarios, we min–max scaled the ARI. It is a simple rescaling method that rescales scores to a range between 0 and 1, allowing for consistent interpretation across methods. The formula used to scale the ARI is


$${\mathrm{ARI}}_{\mathrm{scaled}}=\frac{\mathrm{ARI}-\min(\mathrm{ARI})}{\max\left(\mathrm{ARI}\right)-\min(\mathrm{ARI})}$$

To ensure that both metrics, ARI and ADR, follow the same “higher-is-better” interpretation, we transformed the ADR scores using 1 minus the min–max scaled ADR


$${\mathrm{ADR}}_{\mathrm{scaled}}=1-\frac{\mathrm{ADR}-\min(\mathrm{ADR})}{\max\left(\mathrm{ADR}\right)-\min(\mathrm{ADR})}$$

## 3 Results

### 3.1 CLUEY enables cell clustering and the number of cell type estimation

**Figure 2.**
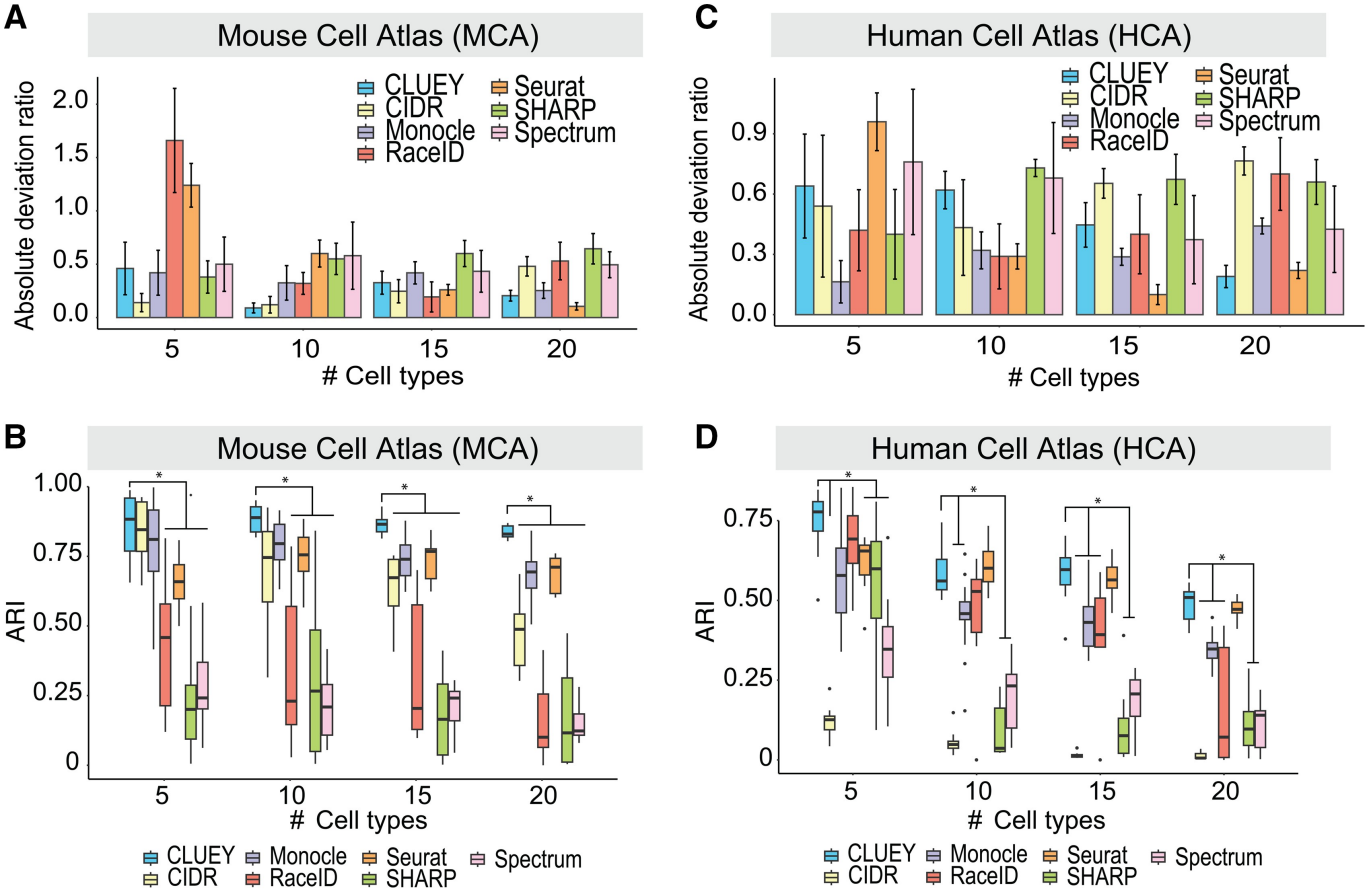
Benchmarking CLUEY on MCA and HCA datasets. (A, C) For each method, the absolute deviation ratio between the estimated number of clusters and true number of cell types for (A) MCA, and (C) HCA. The number of cell types in the query datasets were increased from 5 to 10, 15, and then 20. Error bars represent two standard errors above the means. (B, D) For each method, ARI quantification of cell clustering concordance with predefined cell type labels from for (B) MCA, and (D) HCA. Same as above, the number of cell types in the query datasets were increased from 5 to 10, 15, and then 20. *P < .05 from Wilcoxon rank sum test.

We compared CLUEY’s performance against seven alternative methods using the Mouse Cell Atlas (MCA) and Human Cell Atlas (HCA) datasets. Our evaluation focused on two key areas, including the ability to estimate the true number of cell types (deviation) and the quality of cell clustering, which we evaluate by comparing cluster assignments against known annotations. We note that these comparisons are to evaluate CLUEY on guiding clustering as opposed to comparing different clustering algorithms. We stress that these annotations serve only as a proxy for biological coherence and CLUEY does not require that each cluster map one-to-one to a discrete cell type, as clusters may also capture transitional or mixed-state populations. First, we generated 40 balanced datasets for each atlas by subsampling the data and varying the number of cell types. The query datasets for MCA were sequenced using FACS, while those for HCA were sequenced using 10X Chromium. To assess CLUEY’s generalizability for cross-platform predictions, we generated knowledgebases for CLUEY from both the 10X and FACS-sequenced data and assessed its performance on FACS- and 10X-sequenced query data, respectively. Results from MCA data analyses demonstrated that CLUEY is highly competitive in estimating the number of cell types in the data (Fig. 2A) and lead to highly concordant cell clustering compared to predefined cell type labels from MCA (Fig. 2B). While its performance on estimating the number of cell types in HCA is less striking compared to other methods, the quality of the cell clustering as quantified by ARI remain high across HCA datasets (Fig. 2C and D).

**Figure 3.**
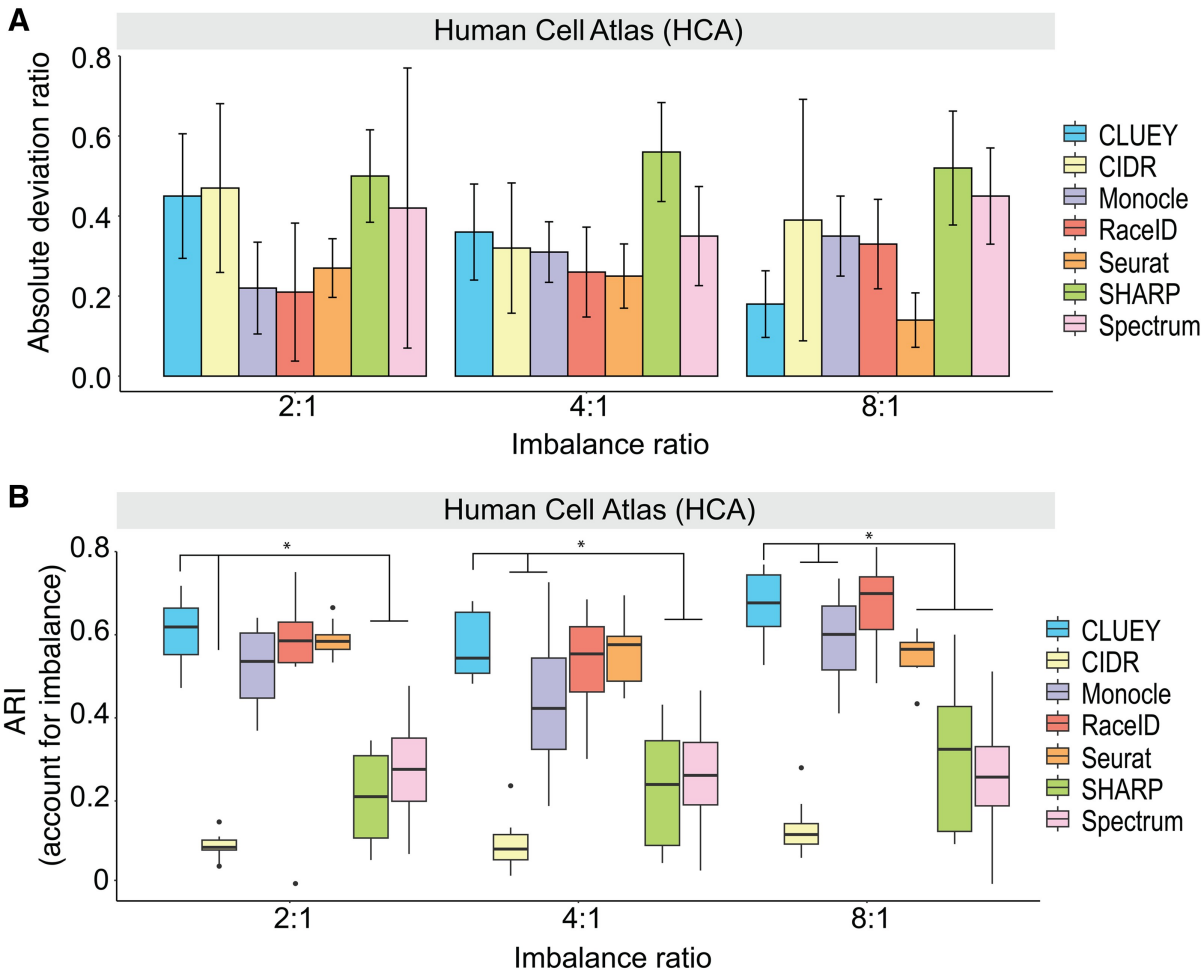
Evaluation of CLUEY and alternative methods on imbalanced data. (A) Absolute deviation ratio on number of cell type estimation for each method on the HCA datasets. *X*-axis represents the imbalance ratio between the major and minor cell types. Thus, an imbalance ratio of 2:1 means there are 200 and 100 cells in the major and minor groups, respectively. Error bars represent two standard errors above the means. (B) ARI quantification of cell clustering for each method on the same HCA datasets as in (A). *P < .05 from Wilcoxon rank sum test.

Next, we also tested CLUEY’s ability to correctly estimate the number of cell types in imbalanced query data. In comparison to alternative methods, CLUEY’s deviation from the true number of cell types remain low and stable across varying imbalance ratios (Fig. 3A). This is largely mirrored by the quality of cell clustering, where CLUEY achieves best performance in most cases across all imbalance ratios (Fig. 3B). It is worth noting that better cell clustering (i.e. higher ARI) does not necessarily lead to a more accurate estimation of the number of cell types (Yu *et al.* 2022).

Overall, these results demonstrate CLUEY’s utility in both cell clustering and cell type estimation.

### 3.2 CLUEY demonstrates robust performance on knowledgebase generated from independent data sources

**Figure 4.**
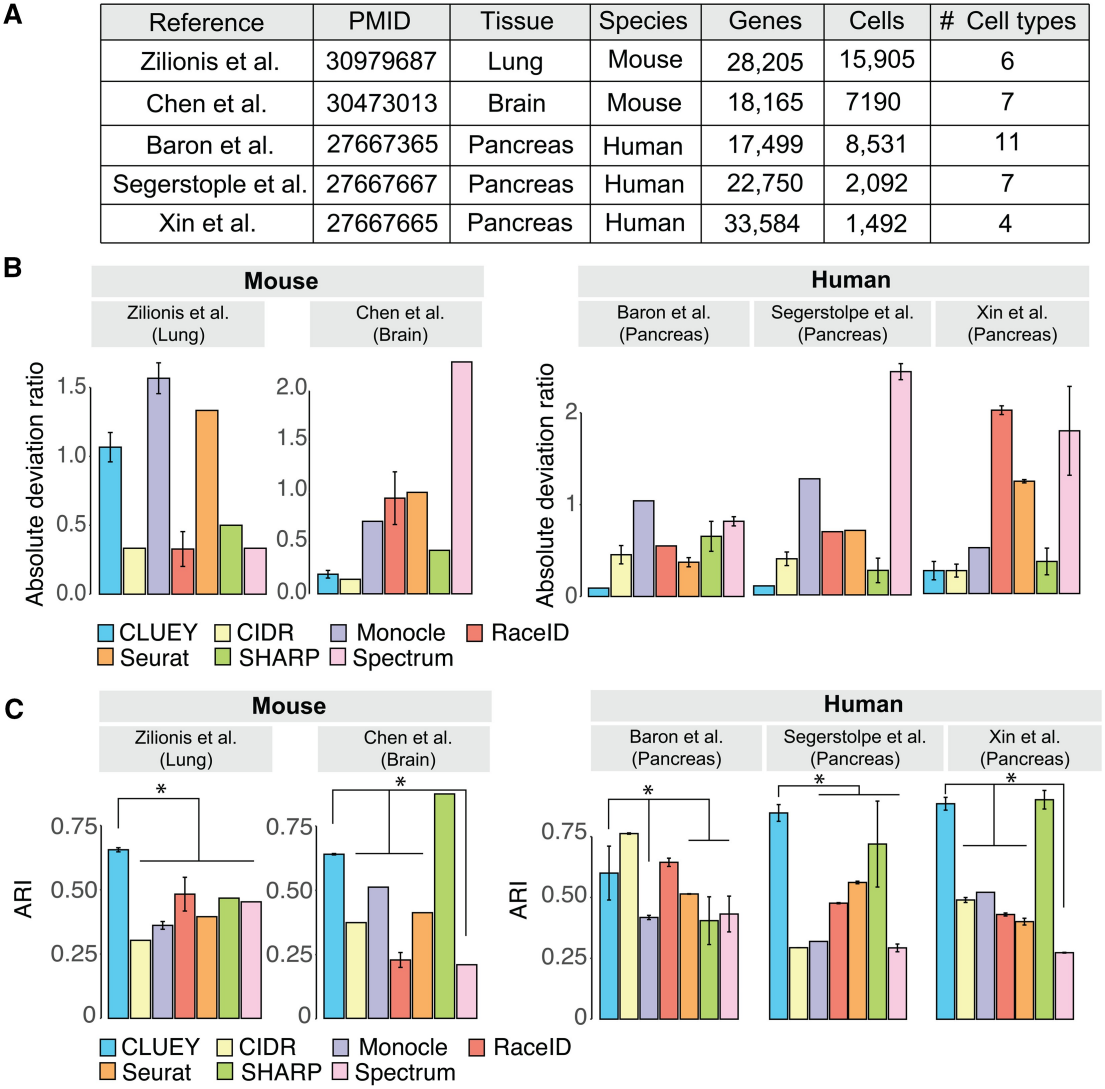
Performance on independent mouse and human datasets. (A) Description of datasets used for independent evaluation. (B) Absolute deviation ratio on number of cell type estimation for each method on the four datasets. Error bars represent two standard errors above the means. (C) For each clustering method, ARI quantification of cell clustering with respect to the original cell type labels from each study. *P < .05 from Wilcoxon rank sum test.

Single-cell RNA-seq datasets are often generated from both human and mouse samples. To evaluate CLUEY’s ability to estimate the number of cell types in independent datasets from both species, we tested CLUEY against alternative methods on four independent datasets from each species datasets (Zeisel *et al.* 2015, Baron *et al.* 2016, Segerstolpe *et al.* 2016, Zilionis *et al.* 2019). These datasets were selected to ensure a varying number of cells and cell types between the two species, including the organs tested (Fig. 4A). Our experiments on these datasets revealed that CLUEY significantly outperforms alternative methods on estimating true number of cell types especially in the Lung and the two Pancreas datasets (Fig. 4B). In terms of cell clustering, CLUEY performed the best in the Lung dataset compared to other methods and show competitive results in the other three datasets (Fig. 4C). It is worth noting that in some cases the number of cell types estimated by SC3 greatly deviate from the true number of cell types, resulting in poor clustering results (e.g. in the Lung dataset). Finally, compared to alternative methods, CLUEY has the advantage of offering interpretable clustering results, as it outputs both statistics and the most similar cell types in the knowledgebase used to guide the estimation process. This information can be utilized to further refine the clusters by guiding the annotation of clusters at a higher resolution, as well be demonstrated in the next section.

### 3.3 CLUEY enables multimodal clustering without the need for a multi-omic knowledgebase

**Figure 5.**
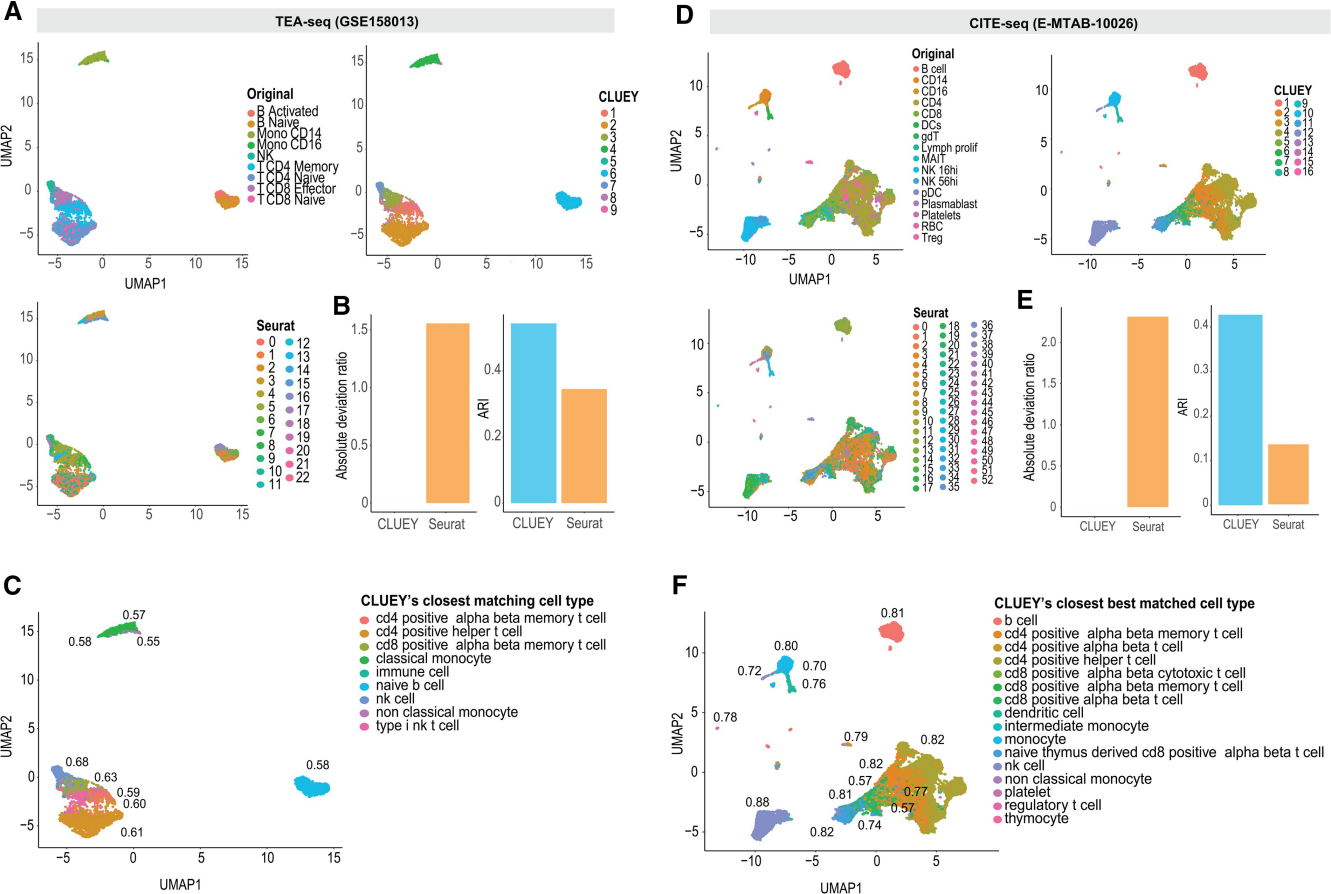
CLUEY enables multimodal clustering and annotation without the need for a multi-omic knowledgebase. (A) UMAPs colored by original cell type labels and clustering output by CLUEY and Seurat for a tri-modality TEA-seq dataset. (B) Barplots of absolute deviation ratio and ARI between the original cell type labels and clustering output of CLUEY and Seurat for TEA-seq data. (C) Closest matching cell types output by CLUEY for TEA-seq data. Numbers next to each cluster are correlation coefficients of the cluster profile with respect to the closest matching cell types. (D) UMAPs colored by original cell type labels and clustering output by CLUEY and Seurat for a bi-modality CITE-seq dataset. (E) Barplots of absolute deviation ratio and ARI between the original cell type labels and clustering output of CLUEY and Seurat for CITE-seq data. (F) Closest matching cell types output by CLUEY for CITE-seq data. Numbers next to each cluster are correlation coefficients of the cluster profile with respect to the closest matching cell types.

To assess CLUEY’s utility and performance in cluster single-cell multi-omics data, we compared it to Seurat across two datasets including the TEA-seq and CITE-seq datasets. In these experiments, we used the knowledgebase generated from HCA 10x data. Our results demonstrated high cluster concordance and accurate cell type annotation by CLUEY compared to the cell type labels from the original studies in both datasets (Fig. 5). In comparison, Seurat appears to overestimate the number of cell types and yield lower ARI values compared to CLUEY (Fig. 5B and D). Notably, while CLUEY also overpredicted the number of clusters in the TEA-seq dataset (Fig. 5A), all clusters corresponded to immune cells, aligning closely with the original cell type labels. For instance, CLUEY grouped cells originally labeled as “Mono CD16” in the TEA-seq data as a single cluster which correlated with the “Mature NK T Cell” in the knowledgebase, and cells labeled as “Mono CD14” were also grouped together and most correlated with “Dendritic Cell” and “Classical Monocyte”, consistent with their known CD14 expression. Conversely, although CLUEY underestimated the number of cell types in the CITE-seq dataset, it accurately grouped cells into clusters that were informed by the related broader cell types. For example, cells labeled as “NK 16hi” and “NK 56hi” in the original data were correctly grouped as a cluster which correlated most with “NK Cell” (i.e. Natural Killer cells), as the finer cell types were not present in the knowledgebase generated from the HCA 10X. These findings underscore CLUEY’s ability to cluster multimodal data without requiring a matched multimodal dataset, expanding its utility as multi-omic datasets become more prevalent.

**Figure 6.**
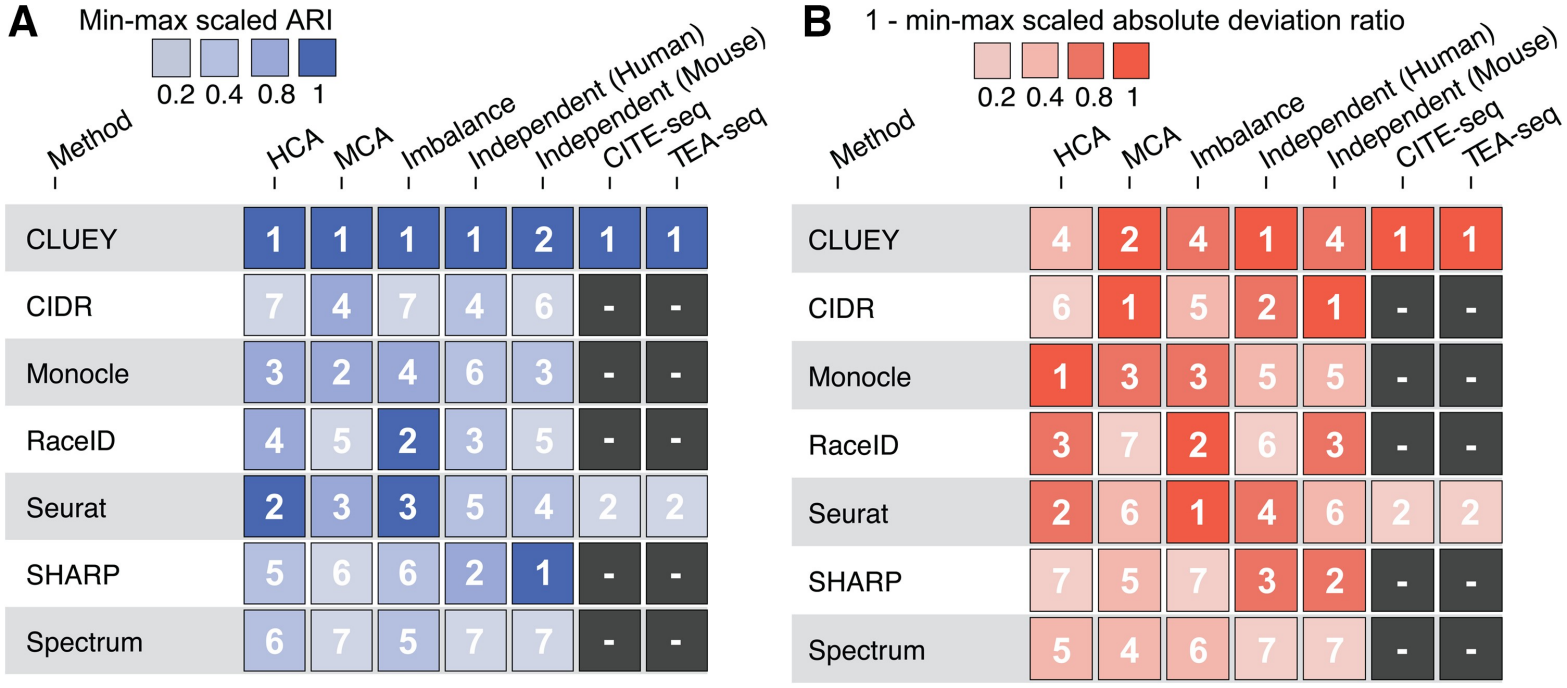
Summary of overall method performance on cell clustering and number of cluster estimation. (A) Heatmap showing the min–max scaled ARI for CLUEY and other methods across seven tested scenarios. (B) Heatmap showing the min–max scaled absolute deviation ratio for CLUEY and other methods across tested scenarios. For ease of interpretation, we transformed the values using 1 minus the min–max scaled absolute deviation ratio, so that higher values indicate better performance.

Finally, we summarize the method performance across all tested scenarios in Fig. 6. These aggregate rankings show that overall CLUEY performs competitively in achieving high ARIs and low ADRs among the methods and outperforms Seurat for multimodal data analyses.

## 4 Discussion

In this work, we have presented CLUEY, a knowledge-guided framework for biologically interpretable clustering. CLUEY employs a novel recursive clustering algorithm and jointly utilizes differentially stable genes to determine the optimal number of clusters while providing the closest matching reference cell types for interpretability. Importantly, CLUEY aggregates each cluster’s cell profiles to reduce data sparsity and amplify shared expression signals, thereby improving clustering stability (Murphy and Skene 2022). To ensure that CLUEY does not over‐depend on knowledgebase, the cluster selection scores it computes combines a data‐driven metric (the explained‐variance ratio, BSS/TSS) and the knowledge‐driven correlation score. Each cluster is annotated with its top reference match and the corresponding Fisher‐Z–transformed correlation coefficient, enabling users to flag clusters with low correlations (e.g. below 0.3–0.4) as “unassigned” for further review. We note that CLUEY’s primary aim is to estimate the optimal number of clusters and propose candidate cell type associations at the cluster level, rather than to assign definitive labels to individual cells. By combining data‐driven and knowledge‐driven criteria, CLUEY produces interpretable, hypothesis‐driven cluster suggestions that support, rather than supplant, expert manual annotation, particularly when novel or poorly matched populations are encountered.

Advances in sequencing technologies recently have enabled us to capture multiple layers of molecular information within individual cells. While single-cell transcriptomics pioneered entry into the single-cell era, it only captures one layer of cellular biology. Complementing transcriptomic data with additional modalities, such as chromatin accessibility by ATAC-seq and surface protein expression by CITE-seq may enhance analyses, including clustering and annotation (Tang *et al.* 2023, Yu *et al.* 2023). In light of this, CLUEY has the capacity to integrate multiple modalities to harness the information within multimodal data and detect the number of cell types using this information. CLUEY enables multimodal data clustering without requiring a corresponding multi-omic knowledgebase. Thus, users have the flexibility of providing their own gene sets using only RNA modality, derived from either uni- or integrated multimodal data, further enhancing its versatility and applicability. Nevertheless, identifying the optimal approach for integrating unmatched multimodal data remains a challenge, and multiple methods have been recently developed for achieving this task (Argelaguet *et al.* 2021, Lee *et al.* 2023). Future work will involve determining an efficient strategy for integrating unmatched multi-omic datasets.

Another challenge is accurately deciphering cell state dynamics. While conventional methods typically categorize cell type identity as discrete states, cells of the same cell type often exhibit heterogeneity (Trapnell 2015). This continuous trajectory of cell states poses a challenge for determining the threshold for clustering cells as belonging to one cell type or the other. Like many methods, CLUEY treats cell type identities as discrete and groups a cell to a cluster which is most correlated to a cell type in the knowledgebase. However, CLUEY provides a score for each cluster based on the correlation coefficient, recognizing that a cell’s identity is continuous and often will not perfectly match the average profile of its most correlated cell type in the knowledgebase. Nevertheless, discrete categorization of cell types simplifies analysis and interpretation but can overlook more subtle molecular variations that occur as cells move from one cell state to another. Thus, it will be important to capture the inherent relationships between genes and how they change within cell states to better identify cell state changes which can further inform the clustering process and lead to more biologically interpretable and accurate clustering.

Beyond cell types and states, it is possible that cells in the query data are not present in the knowledgebase. Some methods define a minimum threshold to designate these cells as unassigned or potentially novel. Yet this parameter is often arbitrarily determined with minimal biological relevance (Aran *et al.* 2019, Kong *et al.* 2022). Given CLUEY is a knowledge-based clustering method, we have opted for the current approach to provide correlation scores associated with the final clusters to provide as much information for further downstream analyses. For example, potential absent or novel cell types can be inferred and further explored using the correlation scores and provided by CLUEY. While a threshold may be easier to interpret, we believe it can bias the conclusions drawn as the appropriate minimum threshold will change depending on many factors, including the sequencing platforms used to generate the data. This approach allows CLUEY to group cells together that are most correlated with a cell type in the knowledgebase but does not strictly assign them to a cell type. Importantly, the final clusters can have heterogeneous correlations scores, allowing users to identify potential novel subpopulations in the data.

In single-cell analysis, “unbiased” clustering methods group cells by molecular similarity and then annotate those clusters, while “biased” classification methods assign each cell a type directly using supervised models trained on reference markers or datasets. In particular, classification methods typically assumes that every relevant cell type is well represented in the reference and that the reference and query share matched feature spaces (e.g. identical genes, peaks, or proteins), limiting its use with heterogeneous query data and multimodal single-cell omics data that do not share identical feature spaces with the references. CLUEY bridges these paradigms by performing unsupervised clustering guided by curated cell identity genes, producing clusters that are both statistically coherent and biologically interpretable. It uses the knowledgebase only to inform clustering, rather than to impose labels directly, and therefore can be applied when modalities differ. Furthermore, CLUEY acknowledges the heterogeneity in the cells by providing cluster‐level annotation with scores that help distinguish well‐matched versus from those that may represent mixed or novel types/states. By combining the exploratory power of unbiased clustering with the interpretability of knowledge-driven annotation, CLUEY complements per‐cell classification tools and offers a novel framework for resolving cellular heterogeneity that can confound direct classification methods.

## 5 Conclusion

In summary, CLUEY is a method that enables knowledge-guided cell type detection and clustering of single-cell omics data. It facilitates the integration and clustering of multi-omic query data without the need for a matching multi-omic knowledgebase and performs a novel recursive clustering algorithm to generate stable and robust results. The knowledge-guided approach implemented in CLUEY marks a step toward biologically-informed clustering and for providing researchers with biologically interpretable clustering, thereby enhancing informed decision-making in subsequent downstream analyses.
